# Persisting olfactory dysfunction in post-COVID-19 is associated with gustatory impairment: Results from chemosensitive testing eight months after the acute infection

**DOI:** 10.1371/journal.pone.0265686

**Published:** 2022-03-23

**Authors:** Constantin A. Hintschich, René Fischer, Thomas Hummel, Jürgen J. Wenzel, Christopher Bohr, Veronika Vielsmeier

**Affiliations:** 1 Department of Otorhinolaryngology, Regensburg University Hospital, Regensburg, Germany; 2 Smell & Taste Clinic, Department of Otorhinolaryngology, TU Dresden, Dresden, Germany; 3 Institute of Clinical Microbiology and Hygiene, Regensburg University Hospital, Regensburg, Germany; Barnard College, UNITED STATES

## Abstract

Olfactory and gustatory disorders are prominent symptoms of acute COVID-19. Although both senses recover in many patients within weeks to months, persistency has been described in up to 60%. However up to now most reports on the course of chemosensitive disorders after COVID-19 are not based on psychophysical testing but only on subjective patients’ ratings. In this study we assessed both olfaction and gustation using psychophysical tests eight months after COVID-19. Validated psychophysical testing revealed hyposmia in 18% and hypogeusia in even 32% of 303 included patients. This shows that olfactory and especially gustatory disorders have to be seen as important chronic symptoms post-COVID-19. The high prevalence of gustatory dysfunction indicates that gustatory function does not recover or might even deteriorate in the months following the acute infection.

## Introduction

The sudden onset of olfactory and gustatory impairment is an important, if not even pathognomonic symptom of an acute SARS-CoV-2 infection [1]. The overall prevalence of olfactory and gustatory dysfunctions is around 50% [2]. Moreover, trigeminal impairment has been associated with acute COVID-19 [3, 4]. Dysfunctions of olfaction and gustation have been reported to recover in many cases quickly within the first weeks [5–7]. However, in other patients recovery takes longer, and in 10–60% of those affected there is little or no recovery during the first months [8–12]. Hence, long-term chemosensory dysfunction (i.e. three months and more after the onset of COVID-19) which cannot be explained by another potential different etiology have to be seen as symptoms of the post-COVID-19 condition [13]. Up to this point, most studies of olfaction and gustation after COVID-19 are based on patient self-ratings [8–11, 14]. This represents a major bias: In both acute COVID-19 [15] and for pre-COVID-19 conditions [16] subjective patients’ self-evaluation does not correlate with the results of validated psychophysical testing.

Until now, still there are only few publications in which valid and reliable olfactory psychophysical tests have been used to assess smell in the long-term after COVID-19, and this is even less so for gustatory function [12, 14, 17]. In the present study, we assessed olfactory and gustatory function eight months after acute COVID-19 both through self-report and psychophysical tests, in order to quantify the prevalence of hyposmia and hypogeusia in post-COVID-19 condition.

## Material and methods

The study was conducted in the Department of Otorhinolaryngology of Regensburg University Hospital in collaboration with the Institute of Clinical Microbiology and Hygiene of Regensburg University Hospital. It was supported by a grant for COVID-19 related research of the *Bavarian* State *Ministry* for *Science* and *Art* and conducted after approval by the ethics committee of the University Regensburg (approval number 20–1766_4–101). The study was performed in accordance with the ethical standards of the Declaration of Helsinki and its later amendments. Detailed information was provided to the patient in written form and their consent was obtained in written form, too.

### Patients

The database of the Institute of Clinical Microbiology and Hygiene of Regensburg University Hospital was screened for patients who were tested positive for SARS-CoV-2 by reverse transcription-polymerase chain reaction (RT-PCR) between 01.03.2020 and 31.08.2020. During that period 2472 positive PCR-tests of 1675 patients were conducted. Out of these patients, 797 were excluded because of fatal disease, age below 18 years, or missing personal data. Eight months after their COVID-19 878 patients were invited to participate in this study by mail. In the case of missing postal addresses, external hospitals and doctors’ offices have been contacted for assistance.

The invitation letter contained the study information, forms for informed consent, and a first questionnaire about patients’ preconditions, the course of the SARS-CoV-2 infection and other details. Overall, 454 patients replied to this first questionnaire either using the attached stamped and addressed envelope or through an online survey. To ensure a high level of participation psychophysical testing was not performed in the hospital but at home. Hence all patients who replied positively to the invitation letter were sent the second letter containing smell and taste tests, detailed instructions, and a second questionnaire. 316 patients responded to the smell and taste test, of which 13 patients had to be excluded due to missing data.

### Patients’ questionnaire

Patients were asked to answer questions on medical preconditions and the course of the SARS-CoV-2 infection. Additionally, they self-rated their subjective olfactory and gustatory function using a visual analogue score from 1 to 10 (1 having no smell/taste and 10 having no impairment in smell/taste).

### Psychophysical testing

Psychophysical testing consisted of validated and blinded tests for olfaction (NHANES Pocket Smell Test, Sensonics, Haddon Heights, NJ, US) and gustation (Taste Strips Test, Burghart Messtechnik, Holm, Germany). An attached instruction manual ensured correct self-administration (Hintschich et al., 2020).

Olfactory function was assessed with the NHANES Pocket Smell Test. This scratch-and-sniff suprathreshold olfactory test comprises eight familiar smells: chocolate, strawberry, smoke, leather, soap, grape, onion, and natural gas. Six correct answers or more indicate normosmia, five and less correct answers indicate hyposmia/anosmia [18].

Gustatory function was tested using the Taste Strips Test. It consists of 16 impregnated filter paper strips to test for four different concentrations of each taste quality (sweet 1: 0.4 g/ml sucrose; sweet 2: 0.2 g/ml sucrose; sweet 3 0.1 g/ml sucrose; sweet 4: 0.05 g/ml sucrose), sour (sour 1: 0.3 g/ml citric acid; sour 2: 0.165 g/ml citric acid; sour 3: 0.09 g/ml citric acid; sour 4: 0.05 g/ml citric acid), salty (salty 1: 0.25 g/ml sodium chloride; salty 2: 0.1 g/ml sodium chloride; salty 3: 0.04 g/ml sodium chloride; salty 4: 0.016 g/ml sodium chloride), and bitter (bitter 1: 0.006 g/ml quinine hydrochloride; bitter 2: 0.0024 g/ml quinine hydrochloride; bitter 3: 0.0009 g/ml quinine hydrochloride; bitter 4: 0.0004 g/ml quinine hydrochloride). Each strip was packed in an individual paper bag, which was numbered in a pseudorandomized order from 1 to 16, to allow a blinded self-administration at home. The attached instructions described the self-administration of the test. Patients were instructed to rinse the mouth with tap water and to observe a break of one minute after each strip. The test was presented as a non-forced choice forced task: Additionally, to the answers “sweet”, “sour”, “salty”, and “bitter” patients could choose “no answer”. This ensured the correct self-administration of the Taste Strips Test and has been validated [19, 20] and successfully utilized [16] previously. The correctly identified strips were summed up to the total Taste Strips score. Normogeusia was defined as 9 or more correctly identified strips [21].

Taste Strips scores were compared to normative data by Welge-Lüssen et al. [16]. Without prior screening, a total of 761 patients were psychophysically tested for their gustatory function using the very same Taste Strips test in a non-forced choice paradigm. The distribution of age (Welge-Lüssen et al.: mean age 35.6 years ± 19.3; this study: mean age 49.0 years ± 14.4) and sex (Welge-Lüssen et al.: 61% female; this study: 55%) were comparable.

### Statistics

Data was analyzed using SPSS Statistics software (version 26, IBM, Armonk, NY, US). Graphs were illustrated using Prism software (version 9, GraphPad Software, San Diego, CA, USA). Unless stated differently values are given as mean ± standard deviation (SD). *p*<0.05 was considered as statistically significant. Continuous data were analyzed for statistical significance using unpaired two-tailed Student’s t-tests. The corresponding homogeneity of variance was assessed using Levene’s test. Proportions between cohorts was calculated using the Z-test. Categorical data was compared between groups using Fisher’s exact test. Correlations were determined using Pearson’s or phi correlation.

## Results

303 patients (mean age 49.0 years ± 14.4) who were tested positive for SARS-CoV-2 by RT-PCR eight months before were included in this study. 167 patients (55%) were female, and 136 patients (45%) were male.

### Subjective rating

70% and 74% of the patients stated retrospectively to have suffered an olfactory and a gustatory impairment, respectively, during the acute phase of COVID-19. Eight months later these figures were at 28% and 24%. In the visual analogue score (VAS) patients self-rated their present olfactory function as 7.9±2.1 and gustatory function as 8.2±1.8. Forty percent indicated the presence of dysgeusia during COVID-19 and 10% at the time of the follow-up.

### Smell test

**Fig 1:**
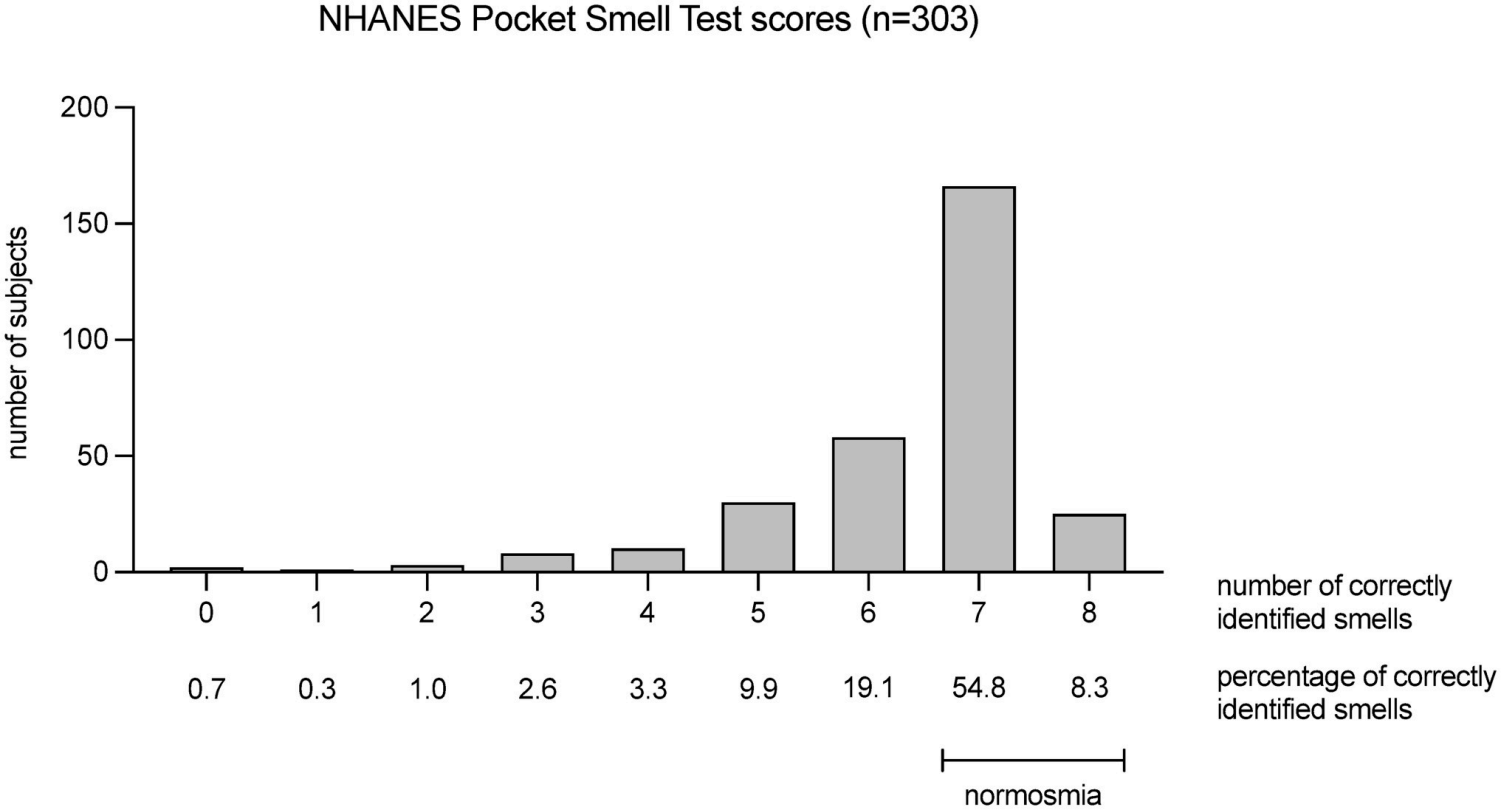
Results of olfactory test. Histogram of the NHANES Pocket Smell Test score as number of correctly identified smells, and percentage of the total study population. Normosmia is defined as six or more correct answers and hyposmia as five and less correct answers [18].

The mean score of the NHANES Pocket Smell Test was 6.4±1.3 with 54 patients (18%) scoring in the hyposmic range (5 or less correct answers), and 249 patients (82%) scoring in the range of normosmia (Fig 1). There was a weak but significant negative correlation between age and smell score (r = -0.19, *p*<0.001).

### Taste test

**Fig 2:**
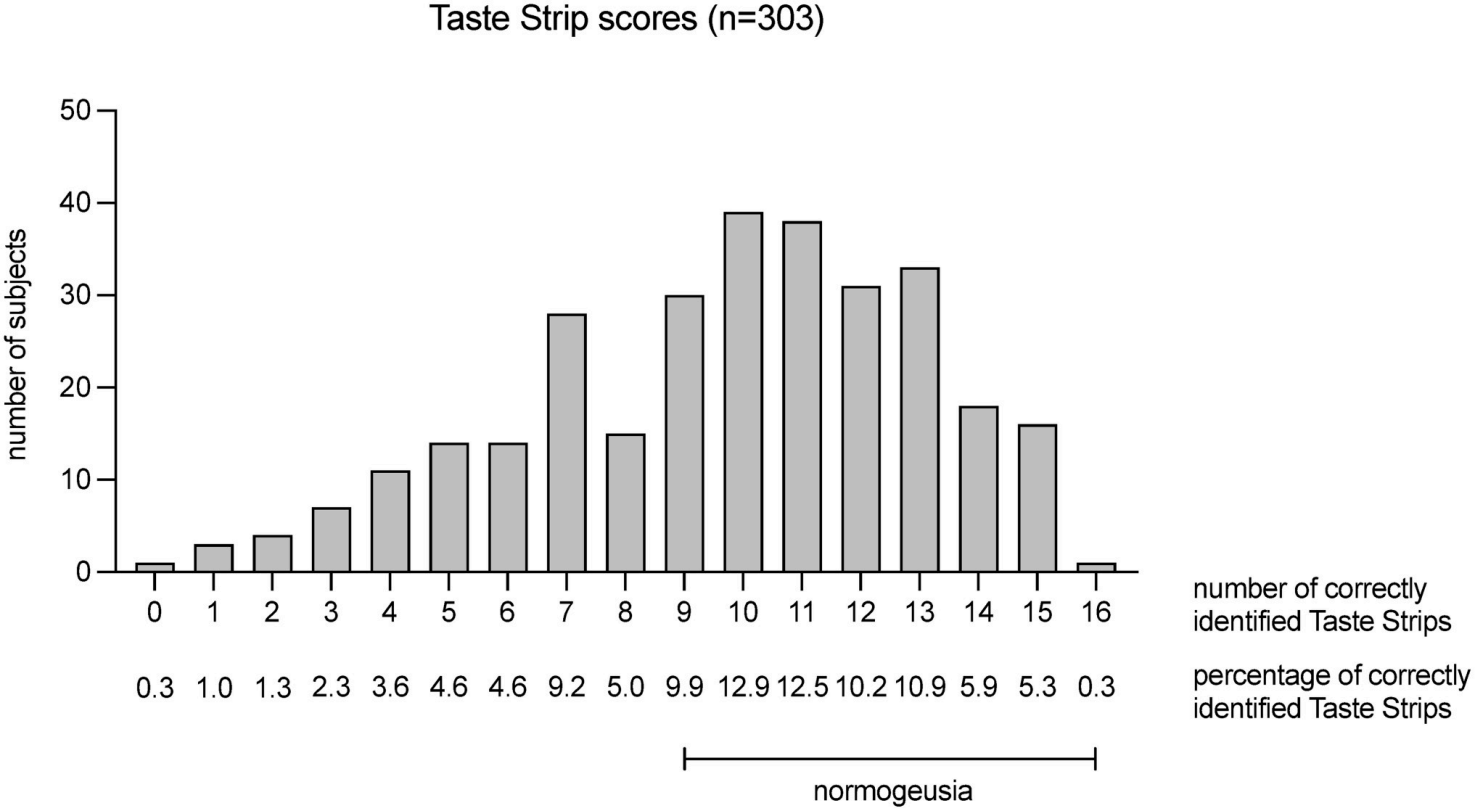
Results of gustatory test. Histogram of the Taste Strips score as number of correctly identified strips, and percentage of the total study population. Normogeusia is defined as nine or more correct answers and hypogeusia as eight and less correct answers [21].

The mean Taste Strips score was 9.7±3.4. 97 patients (32%) scored in the range of hypogeusia (Fig 2); 206 patients (68%) scored in the normogeusic range. Table 1 shows the percentage of correct answers of the single Taste Strips.

**Table 1:** Comparison of the gustatory test between this study and normative data.

	Percentage of correctly identified Taste Strips		
Taste Strip	Results of this study	Normative data form Welge-Lüssen et al., 2011	*p* value
sweet 1	93	98	<0.001
sweet 2	85	97	<0.001
sweet 3	67	91	<0.001
sweet 4	55	87	<0.001
sour 1	78	94	<0.001
sour 2	54	80	<0.001
sour 3	26	58	<0.001
sour 4	5	19	<0.001
salty 1	81	96	<0.001
salty 2	77	91	<0.001
salty 3	47	82	<0.001
salty 4	36	75	<0.001
bitter 1	79	96	<0.001
bitter 2	75	87	<0.001
bitter 3	67	86	<0.001
bitter 4	43	58	<0.001

Correctly identified Taste Strips in this study compared to normative data by Welge-Lüssen et al. [16] (Z-test; note that the higher the number of the Taste Strip the lower its concentration; exact concentrations are provided in the methods section).

More women than men were normogeusic (80% vs. 54%; *p* = 0.001). Women scored also higher than men (10.5±3.0 vs. 8.6±3.4; *p*<0.001). Interestingly, patients who were hospitalized on intensive care units were significantly more often hypogeusic (51% vs. 28%; *p* = 0.003) and also scored significantly lower in gustatory testing than those without intensive care hospitalization (8.3±3.5 vs. 10.0±3.3; *p* = 0.002). Allergies (p = 0.025) and a prior radiotherapy of the head and neck region (p = 0.002) had a significant influence on gustation. Other potential covariates as smoking, prior surgery of the paranasal sinuses, the nasal septum/inferior turbinates, or the brain, chronic rhinosinusitis, neurological diseases, or a prior traumatic brain injury had no significant influence on gustatory function (p>0.05).

### Correlation between smell and taste tests

A highly significant association between tested hyposmia and tested hypogeusia was found (φ = 0.24; *p*<0.001). Correspondingly, a weak, but significant correlation could be demonstrated between the scores of the Pocket Smell Test and the Taste Strips (r = 0.32; *p*<0.001).

### Correlation between subjective ratings and psychophysical results

Self-rated olfactory function (VAS score) correlated moderately with the score of the Pocket Smell Test (r = 0.42, *p*<0.001). However, between self-rated taste function and taste strips score no such correlation could be observed (r = 0.09, *p* = 0.14).

## Discussion

To the best of our knowledge this is the first study to psychophysically assess gustation post-COVID-19. Additionally, it is the largest study to test both olfactory and gustatory function using validated and blinded psychophysical tests in the long-term after COVID-19. In our cohort around 70% of the population stated retrospectively to have experienced hyposmia and hypogeusia, respectively. Eight months later around 25% still reported chemosensory impairments. This magnitude is in line with the reports on persisting subjective chemosensory disorders months after COVID-19 [8–12, 17].

Based on the psychophysical smell test 18% patients were classified as hyposmic. This is not only lower than subjective reports from the present and other studies, but also lower than prior test results: Boscolo-Rizzo et al. found an olfactory impairment in 60% when performing the comprehensive UPSIT six months after COVID-19 [14]. The different follow-up period as well as the lower prevalence of psychophysically confirmed hyposmia in our study can possibly be explained through the sensitivity of the utilized test. Previously, a limited test sensitivity of 60% has been identified for the NHANES Pocket Smell Test [18]. Hence, the prevalence of hyposmia could be underestimated in the present study. The discrepancies between the various studies may also point towards selection bias in terms of the inclusion of participants.

In contrast, gustatory testing revealed a high prevalence of taste disorders: 32% of our cohort of more than 300 patients were tested hypogeusic (Fig 1). Importantly, the prevalence of hypogeusia after COVID-19 was significantly higher than in the general population: In a healthy sample of 761 patients only 5% were tested hypogeusic using the same Taste Strips Test [16]. Other studies found taste impairment in 15 to 20% of the pre-COVID population [22–24]. Scores for all individual taste qualities (sweet, sour, salty, and bitter) were significantly impaired compared to the results from Welge-Lüssen et al. (all *p*<0.001; Table 1). Gustatory testing showed a lower mean Taste Strips score for sour (1.6±1.0) compared to the three other taste qualities (sweet: 3.0±1.1; salty: 2.4±1.1; bitter: 2.7±1.3). It is notable that the subjects also displayed impaired sensitivity to the other taste qualities as well, relative to the normative data of Welge-Lüssen et al. (sour 2.5; sweet 3.7; salty: 3.4; bitter: 3.3). This suggests that gustatory impairment in post-COVID-19 probably does not specifically affect individual taste qualities.

Interestingly, we found a difference in the prevalence of hypogeusia dependent on the severity of COVID-19. In patients experiencing a mild course hypogeusia could be diagnosed in 28%. However, after intensive care hospitalization due to COVID-19 patients were hypogeusic in even 51%. This finding contrasts with prior publications on gustatory function during acute COVID-19: Various studies found gustatory as well as olfactory dysfunction to be less frequent in patients receiving intensive care hospitalization compared to patients with a mild course of COVID-19 [25–27]. The high prevalence of gustatory dysfunction after intensive care hospitalization could be explained differently. A higher viral load in severe COVID-19 could exacerbate a potential SARS-CoV-2-associated direct damage of the gustatory pathway. Some drugs are known for their long-lasting side effects on taste [28, 29]. And the reduced general condition after severe COVID-19 [30] as well as a reduced neurological outcome after acute respiratory distress syndrome (ARDS) could also further impair gustation [31].

In contrast to olfactory dysfunction [6, 7, 12, 14] the prevalence of psychophysically confirmed hypogeusia might be higher post-COVID-19 than during the acute infection. Three studies assessed taste during acute COVID-19 using the Taste Strips test in a forced-choice paradigm. They found hypogeusia in only 12% to 26% [7, 32, 33]. While the discrepancy between the present results and previous reports may be explained by the test modes (forced-choice testing vs. non-forced choice testing), still the present results obtained eight months after the infection show that taste function does not recover or may even deteriorate in the months following COVID-19. Hence, chronic gustatory dysfunction in more than 30% of the subjects may reflects direct damage of taste buds, indirect inflammatory effects on the gustatory pathway [3], or changes in central processing of taste.

For chronic non-COVID-19 hyposmia a secondary gustatory impairment has been suggested [34–37]. As the processing of chemical information from olfaction, gustation, and trigeminal input uses overlapping brain regions and amplifies each other, a secondary hypogeusia could be explained through reduced amplification of gustation on a central nervous level [38–40]. Prolonged olfactory dysfunction after COVID-19 may also be associated with secondary hypogeusia as it has been shown for pre-COVID hyposmia of various etiologies [37]. Similarly, Huart et al. found significantly decreased gustation in hyposmic COVID-19 patients two weeks after COVID-19 [41].

Two more results of this study support this hypothesis: Firstly, gustatory dysfunction significantly affects any of the four tested qualities. This is in accordance with previous findings of Gudziol et al., showing that anosmic patients have higher thresholds for the gustatory qualities sweet, sour, salty, and bitter [34]. Secondly, in this study psychophysically confirmed hypogeusia was significantly associated with tested hyposmia. Also, the score of NHANES Pocket Smell Test and the Taste Strips Tests correlated significantly with each other. This could not be observed in acute COVID-19 [42] and suggests that a chronic post-COVID-19 hyposmia might secondarily impair gustatory function.

## Conclusion

This study confirmed that olfactory dysfunction can be a chronic symptom even eight months after COVID-19. Interestingly, hypogeusia, a rare symptom during the acute infection, has been psychophysically confirmed in 32% of the study population. This must be seen as a frequent symptom of post-COVID-19 condition and might be partly due to a decreased central nervous amplification.

## Supporting information

S1 Data (XLSX)
